# COVID-19 Vaccine in Inherited Metabolic Disorders Patients: A Cross-Sectional Study on Rate of Acceptance, Safety Profile and Effect on Disease

**DOI:** 10.3390/ijerph191912227

**Published:** 2022-09-27

**Authors:** Albina Tummolo, Annamaria Dicintio, Giulia Paterno, Rosa Carella, Livio Melpignano, Donatella De Giovanni

**Affiliations:** 1 Department of Metabolic Diseases, Clinical Genetics and Diabetology, Giovanni XXIII Children Hospital, Azienda Ospedaliero-Universitaria Consorziale, 70126 Bari, Italy; 2 Medical Direction, Giovanni XXIII Children Hospital, Azienda Ospedaliero-Universitaria Consorziale, 70126 Bari, Italy

**Keywords:** adverse effects, COVID-19, hereditary metabolic disorders, vaccine

## Abstract

Background: Vaccines for COVID-19 have had a significant impact on the spread of COVID-19 infection, reducing the incidence and mortality of the infection in several countries. However, hesitancy toward this vaccine is a global health issue for the general population The Vaccine acceptance rate among patients affected with inherited metabolic disorders (IMD), as well as safety profile, has not been described. Methods: We conducted a cross-sectional study, based on a telephone survey, investigating the COVID-19 vaccination rate, the incidence and type of adverse effects (AEs), the reasons for vaccine refusal and the effects on the underlying disease in a cohort of IMD patients followed at a single center and invited directly to vaccination by specialistic team. Results: Seventy-four patients were included in the study, the median age was 23.4 years (min 12.1–max 61.7), 47% (*n* = 85) were females and 61% (107) were affected from impaired metabolism of phenylalanine. By October 2021, 94% (*n* = 163) of them had received at least one dose of the vaccine, which was, in 98% of cases, mRNA-based vaccine, given at the referral hospital in 65% of cases. Overall, 72% of patients with IMD reported AE to the vaccine: 60% after the first dose, 81% after the second. The highest rate of adverse events at the first dose was reported in patients with amino acids related disorders other than impaired phenylalanine metabolism (PKU/HPA) (88%). For the second dose, the PKU/HPA group reported the highest rate of AEs (89% of cases). There was no effect on the underlying disease or acute decompensation after the vaccine. Eleven patients (6%) were not vaccinated because they considered it dangerous. Conclusion: Among individuals with IMD, the vaccination rate was high, the incidence and severity of AEs were comparable to those in the general population with no effects on the disease. Direct contact with the specialist medical team, has proven to reassure patients and effectively contrast hesitancy.

## 1. Introduction

Vaccines represent one of the most effective measures in public health; many infectious diseases have been effectively reduced, eliminated, or prevented through the use of vaccinations over the past century [1]. Vaccines are estimated to prevent up to 3 million deaths each year worldwide [1,2].

Since late 2020, the COVID-19 vaccine has become available in many countries and has been administered to millions of people around the world [3,4]. Vaccination is showing a satisfactory safety profile in the general population as demonstrated by several clinical trials [5,6,7,8]. However, the safety and efficacy of COVID-19 vaccines in subpopulations, such as children, pregnant and lactating women, have not yet been fully studied, although many reports describe comparable efficacy and safety in healthy subjects [9,10].

Patients with rare and chronic diseases have also been invited to be vaccinated by European Organizations [11,12]. However, a number of patients affected by these diseases remain unvaccinated, as the uncertainty for vaccinations seems to also affect patients with rare diseases [13,14,15].

In Italy, vaccination was offered to all patients suffering from genetic and/or chronic diseases from March 2021 onward [16]. From May 2021, patients 12 years of age and older could also be vaccinated [17]. It was free in Puglia, where it was managed in the reference Hospitals after direct contact with specialist reference teams.

Patients with Inherited Metabolic Diseases (IMD) have been involved in this campaign, as COVID-19 vaccines are not contraindicated in this patient group, including those who have had COVID-19 infection [18].

To date, there is a lack of data on COVID-19 vaccination in these patients and, more importantly, the effect of vaccination on the disease, which is a concern for patients with IMD, has not been reported. In our study we aimed to investigate the COVID-19 vaccination rate and the incidence and type of adverse effects (AEs), as well as the reasons for vaccine refusal and the effect on disease, among IMD patients followed in a single center.

## 2. Materials and Methods

### 2.1. Study Design

This is a cross-sectional study conducted on patients followed-up for IMD at a single specialist center in Southern Italy (reference center for the Regions of Puglia and Basilicata). Patients 12 years of age and older were identified through the hospital’s patient database and were contacted by telephone. The Interviews were conducted between September and October 2021 by the specialist team involved in the follow-up of this group of patients.

The following characteristics were recorded: demographic information, including age, sex, underlying disease, comorbidity and previous COVID-19 infection or post COVID-19 vaccine infection; information on vaccination against COVID-19, including first dose +/− second dose, when and where they were given and type of vaccine; information on AEs, including type of reaction, need for medical referral and possible treatment; and information on the effect of the vaccine on the management of the underlying disease, including acute decompensation, altered biochemical parameters (levels of ammonia, phenylalanine and homocysteine), change in the normal treatments regimen and missed scheduled visit. Based on the survey results, we compared the rate of systemic reactions observed in our population after the first and second dose with those reported by other studies conducted in the general population.

In case of no vaccination, the reason for the refusal was investigated through the following open questions: belief that the vaccine would be dangerous, fear of possible vaccine consequences, fear of effects of the vaccine on disease, previous COVID-19 infection or other reasons (e.g., allergies, comorbidities, previous vaccine reaction or other concomitant vaccinations).

All patients were asked to administer the vaccine at the referral hospital, contacted by the referring physician, who administered the vaccine. LSD patients with ongoing enzyme replacement therapy (ERT) were advised to administer the vaccine at least two days after the ERT injection.

This study was approved by the local ethic committee. Verbal informed consent was obtained from all participants and, in the case of subjects between the ages of 12 and 18 years, from both participants and parents.

### 2.2. Statistical Analysis

Continuous variables were expressed as mean (±standard deviation, SD) or median and minimum–maximum value, depending on their distribution; categorical data were expressed as frequency and percentage, and the Chi-squared test was used to compare the two groups. Differences in continuous variables between groups were compared using a Student’s *t*-test for normally distributed parameters, or a non-parametric Mann–Whitney U Test in case of non-normal distribution. Statistical analysis was performed using Microsoft^®^ Excel 2016 MSO, version 2206.

## 3. Results

### 3.1. Study Population

A total of 192 IMD patients were contacted by telephone. Of these, 12 were not available by phone or refused to participate in the study. Six patients provided fragmentary or incomplete information and were excluded. Finally, 174 patients were included in the study (power 0.86, alfa error 0.05). Their median age was 23.4 years (min 12.1–max 61.7), of which 47% (*n* = 85) of them were females (Table 1)

All had regular follow-ups at our specialist center, laboratory monitoring of biochemical parameters, and clinical follow-ups were not interrupted. Most (61%, *n* = 107) had a diagnosis of Phenylalanine metabolism alteration (hyperphenylalaninemia/phenylketonuria—HPA/PKU); a female PKU patient was administered with the COVID-19 vaccine while breastfeeding. The remaining 39% of patients, were affected by other IMD, individual groups of conditions are shown in Table 1. Ninety percent (*n* = 162) lived in Puglia, while 18 (10%) lived in another Italian region.

Nineteen patients (11%) had contracted COVID-19 infection, fourteen (8%) prior to the first vaccine dose, from January 2021; their clinical course was included in our previous paper [19]. Four patients (2%) tested positive for COVID-19 infection between the first and the second scheduled dose of vaccine; in one case, positivity was detected concurrently with the first dose. The clinical course was, in all cases, mild and characterized by chills and/or headache. In these cases, the second dose was not administered, according to the technical information of the vaccine [20].

### 3.2. Vaccination Rate

By October 2021, 94% of our patients received at least one dose of COVID-19 vaccine and 87% received both doses (Table 2).

The second dose was not given for the following reasons: COVID-19 infection after the first dose (*n* = 4) or second scheduled dose after our interview (*n* = 8).

Ninety-eight percent of patients received an mRNA-based vaccine: 152 (93%) received Pfizer Biontech (BNT162b2) vaccine and 8 (5%) received Moderna (mRNA-1273) vaccine.

Less administered was the adenovirus vaccine (Astra-Zeneca), as reported in Table 2. Two patients did not remember the type of vaccine received. In all cases of full course, with known type of vaccines, the same type was administered for both doses.

In 107 patients (65%), the vaccine was administered at the referral hospital. The reasons for the refusal were: distance from the center and/or concomitant infections. No immediate reactions were detected in any vaccinations administered. The vaccine was administered by the general practitioner or family pediatrician in the remaining number of patients. Even in these cases, no immediate reactions were reported.

### 3.3. Adverse Event Rate and Disease Effects

Overall, 118 of 163 with IMD patients (72%) reported AEs to the vaccine: 99 after the first dose (60%), 123 after the second (81%) and 52 after both vaccination doses (34%). Table 3 reports the vaccination rate and AE according by type of conditions.

The highest rate of AEs at the first dose was reported by patients with other amin acids-related disorders (OARD) (88%), 7/8 of them experienced systemic reactions. However, medical referral, in this group, was never reported. AEs were always treated at home with paracetamol or non-steroidal anti-inflammatory drugs (NSAIDs). Within the subgroup of urea cycle disorders (UCD), only two subjects (16%) reported AEs, in both cases presenting pain from local injection, not requiring medical advice (Table 3).

For the second dose, the PKU/HPE group reported AEs in 89% of cases, requiring treatment in 21 of them (21%). The most common systemic AE was fever, reported in 80% of cases (Table 3). The PKU woman, who was breastfeeding at the time of vaccination, received the first dose one month after giving birth. The vaccine was administered at the reference hospital and had no reaction after the first dose. Local pain at the injection site after the second was reported and managed with paracetamol for 48 h. Moreover, for the second dose, the subgroup of patients with UCD reported fewer AEs than others (36%), requiring paracetamol in one case (Table 3).

Effects on underlying disease were reported by one patient (0.6%); the girl had PKU and reported increased Phe levels after the second dose: mean values ± SD before vaccine (six dosages), 468.6 ± 121 µmol/L; mean ± SD values after vaccine (six doses):,567.6 ± 79 µmol/L (*p*-value: 0.15). There was no increase in ammonia levels, no change in diet and/or therapy were made due to AE after the vaccine. No scheduled visits were cancelled or postponed due to the vaccine.

### 3.4. Type of Adverse Events

**Figure 1:**
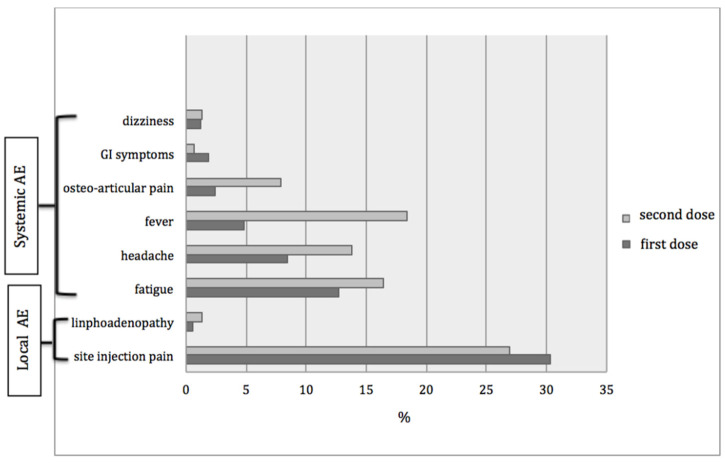
Rates and types of AEs after the first and second dose of COVID-19 vaccine.

After the first dose, injection site pain was the most common local AE, while fatigue and headache were the most frequent of systemic reactions (Figure 1)

For the second dose, local pain remained the most frequently reported AE, while fever and fatigue were the most frequently reported systemic reactions.

Overall, systemic reactions were reported in 68.9% of cases, and local reactions in 33% of cases.

**Figure 2:**
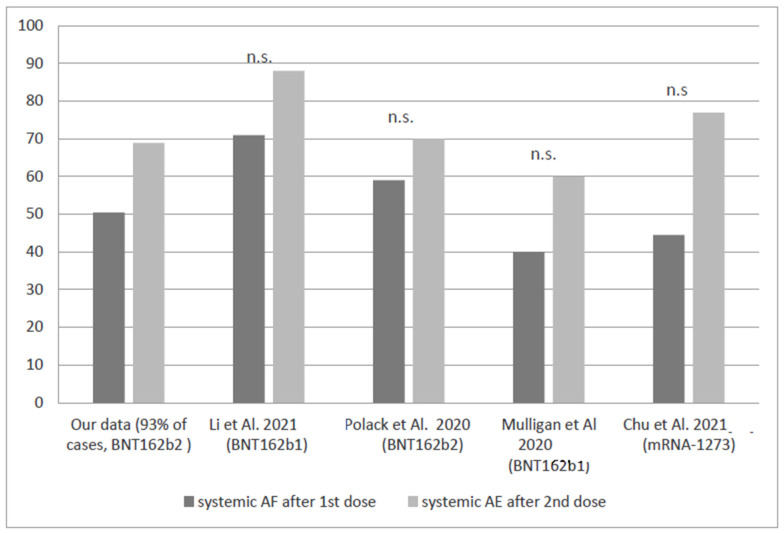
Prevalence of systemic AEs in our cohort and general population as reported by several studies: no significant differences were found between our data and data from other studies for both doses [22,23,24,25].

Comparing the rate of systemic reactions after the first and the second dose in our population with those reported by studies on healthy young adult subjects (considering the median age of our population) [21,22,23,24] (Figure 2) no statistically significant differences emerged between both doses in all cases (lowest *p*-value: 0.89).

### 3.5. Vaccination Refusals

Eleven patients (6%) received no vaccine dose at the time of interview, six females and 5 males, with median age of 27.1 years (min 12.5–max 34.7). Of these, eight (72%) were affected by HPE/PKU, one (9%) by congenital disorders of glycosylation (CDG), one (9%) by OARD, (Homocystinuria) and one (9%) by UCD (Table 3). The reasons for not vaccinating against COVID-19 were: belief that the vaccine would be dangerous (six patients), that it could have effects on disease, worsening of the clinical condition (two cases), fear of the vaccine due to comorbidity (two cases) and other concomitant vaccination (one case) who, however, would have considered COVID-19 vaccination in the near future.

## 4. Discussion

As of early March 2022, more than 880 million doses of vaccines have been administered to people in the EU and the European Economic Area [25]. Although licensed COVID-19 vaccines are declared safe and effective, by the European Medicine Agency, concerns about COVID-19 vaccines mainly relate to the new technology used to manufacture some of the approved vaccines, but also the idea that vaccination can cause adverse effects [14]. In the context of rare diseases and, more specifically, of inherited metabolic disorders, doubts also concern the possible effects that this approach could have on underlying genetic disease, in particular on those at risk of decompensation.

On March 2021, MetabERN experts recommended that IMD patients receive vaccination against COVID-19, when they were offered this opportunity. All IMDs should be considered a top priority for COVID-19 vaccination, particularly those patients at high risk of acute metabolic decompensation, respiratory or cardiac complications, and frequent exacerbation [18].

The COVID-19 pandemic has shown, for the first time, the effects of a worldwide phenomenon on this group of rare disorders, showing that, while , on the one hand, the infection did not produce dramatic consequences on the course of IMDs in the vast majority of cases [19,26], on the other hand, children with IMDs, their families [27] and clinicians involved in their management [28,29] had important and specific needs, unmet during the COVID-19 pandemic.

In this paper, we report that by October 2021, 94% of our patients received at least one dose of the vaccine, which was an mRNA-based vaccine, in almost all cases.

Studies on vaccine acceptance and safety in rare disease are scarce and, to our knowledge, this is the first study COVID-19 vaccine in IMDs.

In reports of COVID-19 vaccination in chronic conditions, the acceptance rate is influenced by the period in which the investigations were conducted. A recently published survey by Tsai et al. [30] studied vaccine hesitation in a cohort of patients with cancer, autoimmune diseases and other comorbid conditions. By February 2021, 25% had received at least one COVID-19 vaccine injection. After the first injection, 69% self-reported local and 40% systemic reactions, which increased after the second injection to 76% and 67%, respectively.

JK Gordon et al. between April and May 2021, studied the effects of the COVID-19 vaccine on a cohort of patients with systemic sclerosis; 75% received at least one dose of the vaccine and 38% received two vaccine doses, at the time of the survey. Adverse reactions were reported after the first dose in 39% of participants and after the second dose in 58% [31]. In this context of disorders, most of the AEs were not serious and did not require hospital admission [32].

Our survey was conducted between September and October 2021, that is, six months after the start of the vaccination campaign for frail patients in Italy [16] and subsequently to other reports. However, the high acceptance rate may be also secondary to the fact that patients were directly invited to receive the vaccine by the referral team and the vaccine was administered at the referral center. It has been reassuring for patients and families and has increased the acceptance rate.

Although we do not have sufficient data to suggest that one vaccine is more suitable for any specific IMD than another, the results of our study support the safety of COVID-19 vaccines based on mRNA technology in this patient group. The vast majority of our sample received the Pfizer Biontech BNT162b2 vaccine. Comparing the systemic effects rate of our data with the clinical trial results of Pfizer BNT162b1/BNT162b2 and Moderna mRNA-1273 vaccines in healthy young adults, we found a comparable prevalence of systemic AEs after both doses as shown in Figure 2, in line with other reports on chronic conditions [30,31].

Our results also pointed out that the vaccine was not able to raise ammonia levels, nor was it a cause of decompensation in the subgroup of IMD with decompensation risk. In fact, for the two cases that reported laboratory changes after the vaccine, the influence of other concomitant factors occurring during that period and influencing those data, cannot be excluded. The vaccine has also demonstrated a safety profile in PKU breastfeeding woman, as reported for the general population [33].

Vaccination in patients with IMD has been a subject of discussion in recent decades, as some metabolic experts still hesitate to vaccinate patients with IMD for fear of the risk of acute decompensation and serious adverse events related to the administration of the vaccine. Some studies report sub-optimal vaccination coverage among IMD patients compared to healthy peers [34], others report the same immunization schedule between IMD children and healthy infants [35].

According to recent evidence, immunization in this patient group was not associated with an increased risk of serious adverse events [36]. Regarding IMD with risk of decompensation, Morgan et al. [37], report that childhood vaccinations were unable to trigger episodes of hyperammonaemia in children with UCDs. Accordingly, in another study on 271 vaccinated children with IMD, the vaccinated subjects did not appear to be at increased risk for serious adverse events [38]

Our study has some limitations, being a cross-sectional study based on telephone interviews, conducted over a month-time and not at the end of the vaccination campaign. Although we have used this method, instead of online questionnaires to reduce self-reporting bias, this may not be completely ruled out. The severity of reported AEs also cannot be objectified, although AE leading to referral to specialist physicians and/or hospitalization can be excluded as all patients were under close clinical supervision by the center. We do not have a control group of people without IMD, although comparison with other studies have shown that AEs are largely similar in type and frequency.

On the other hand, this study has also some strengths, as it represents the only available evidence on the COVID-19 vaccine in patients suffering from different types of IMD, both with and without decompensation risk.

## 5. Conclusions

Among individuals with inherited metabolic disorders, the rate and the severity of AEs were comparable to that of the general population with no effect on the disease. The acceptance rate was high, as direct contact with the specialist medical team on the COVID-19 vaccine was shown to counteract hesitation. This approach should be intensified as misinformation about COVID-19 vaccines also involves patients and families with rare diseases.

## Figures and Tables

**Table 1:** Demographic characteristics of patients and diseases.

	Number	Percentage
Total patients contacted	192	
Total responder patients/available info	174	90
Female gender	85	47
Median age (min-max values)	23.4 (12.1–61.7)	
Diseases		
HPA/PKU	107	61
OAD	16	9
LSD	7	4
UCD	12	7
C-FAOD	12	7
OARD	10	6
CDG	2	1
OTHER IMDs	8	4

HPA/PKU (hyperphenylalaninemia/Phenylketonuria); OAD (Organic acids related disorders); LSD (lysosomal storage disorders), UCD (urea cycle disorders), C-FAOD (carbohydrate-fatty acids oxidation disorders), OARD (other amino acids related disorders); CDG (congenital disorders of glycosylation), OTHER IMDs (5,10-methilenetetrahydrofolate reductase deficiency, type 1 osteogenesis imperfecta, type 1 congenital generalized lipodystrophy).

**Table 2:** Information on COVID-19 vaccination.

	Number	Percentage
**Tot 174**		
**Fully vaccinated (with second dose)**	151	87
**Partially vaccinated (only one dose)**	163	94
**Not vaccinated**	11	6
**Previous COVD19 infection**	14	8
**Subsequent COVID19 infection**	5	3
**Tot 163**		
**Type of vaccine**		
**Pfizer Biontech**	152	93
**Moderna**	8	5
**Astra-Zeneca**	1	0.6
**Do not remember**	2	1
**Vaccine administered at referral Hospital**	107	65
**Adverse reaction to**		
**first dose**	99	60
**second dose (tot 151)**	123	81
**both doses (tot 151)**	52	34

**Table 3:** Effects of the first and second dose of the COVID-19 vaccine based on the type of conditions.

			Effects to First Dose				Effects to Second Dose, *n* (%)				
Disease, *n* of Patients	Median Age	Not Vaccinated *n* (%)	Number/ Rate of Aes	Medical Referral, *n* (%)	Necessity of Treatment	Drug Used	Number/ Rate of AEs	Medical referral, *n* (%)	Necessity of Treatment, *n* (%)	Drug Used	Effect on Disease
HPE/PKU 107	22 (12–51)	8 (7)	73 (73)	3 (3)	8 (8)	paracetamol, NSAIDs, corticosteroids	89 (89)	3 (3)	21 (21)	paracetamol	1 increase in Phe levels
OAD 16	21 (13–39)	0	3 (19)	0	1 (6)		5/13 (38)	1 (8)	2 (15)	paracetamol	none
UCD 12	20 (12–43)	1 (8)	2 (16)	0	1 (9)	paracetamol	4 (36)	0	1 (9)	paracetamol	none
C-FAOD 12	25 (12–42)	0	6 (54)	0		paracetamol	5 (45)	0	1 (9)	paracetamol	none
OARD 10	21 (15–53)	1(10)	8 (88)	0	3 (33)	paracetamol, NSAIDs	8 (88)	0	2 (22)	paracetamol	none
LSD 7	20 (15–49)	0	5 (71)	0	0	none	4 (57)	0	1 (7)	paracetamol	none
OTHER 10	21 (12–61)	1(10)	3 (33)	0	1 (11)	none	8 (88)	3 (33)	4 (44)	paracetamol NSAIDs	none

NSAIDs: non-steroidal anti-inflammatory drugs

## Data Availability

Not applicable.

## Abbreviations

Coronavirus 2019 (COVID-19); inherited metabolic disorders (IMD); adverse effects (AEs); hyperphenylalaninemia/phenylketonuria (HPA/PKU); urea cycle disorders (UCD); OAD (Organic acids related disorders); LSD (lysosomal storage disorders); UCD (urea cycle disorders); C-FAOD (carbohydrate-fatty acids oxidation disorders); OARD (other amino acids related); CDG (congenital disorders of glycosylation), NSAIDs: non-steroidal anti-inflammatory drugs.
